# Prevalence and cost of sickle cell disease in France: real-world analysis using data from the Echantillon Généraliste des Bénéficiaires

**DOI:** 10.3389/fpubh.2023.1215605

**Published:** 2023-09-21

**Authors:** Maud Beillat, Isabelle Durand-Zaleski, France Pirenne, Stève Bénard, Louis Chillotti, Frédéric Galacteros

**Affiliations:** ^1^Pfizer France, Paris, France; ^2^Assistance Publique Hôpitaux de Paris, Paris XII University, Paris, France; ^3^Etablissement Français du Sang – Ile de France, Paris, France; ^4^stève consultants, Oullins, France; ^5^Henri-Mondor Hospital, Assistance Publique Hôpitaux de Paris, Paris, France

**Keywords:** real-world, epidemiology, cost, sickle cell disease, claims, database

## Abstract

Sickle cell disease (SCD) is a genetic disorder of the hemoglobin resulting in chronic anemia, hemolysis, and vaso-occlusions. Its treatment mostly relies on hydroxycarbamide, transfusions, and stem cell transplantation. This study aimed at describing the epidemiology and management of SCD in adolescent and adult patients in France. This was a retrospective study performed among SCD patients aged ≥12 years between 2016 and 2018 and controls. SCD patients were matched on a 1:3 ratio with a group of individuals with no diagnosis of SCD, referred as control group. The matching of SCD patients and controls was a direct matching based on age, sex, CMU-c status (which corresponds to free-of-charge complementary coverage for people with low resources) and geographical region of residence. SCD patients and their matched controls were followed-up for the same amount of time by adjusting controls’ follow-up period to that of the associated patients. This study used claims data from the French representative 1/97th sample of health data system. The main outcomes were the patients’ characteristics and treatments received, healthcare consumptions and related costs among SCD cases and controls. Between 2016 and 2018, 151 patients with ≥6 months of follow-up were identified out of the total population of 732,164 individuals. SCD prevalence extrapolated to the entire population [95% CI] was 19,502 [19,230, 19,778] in 2018. The median (Q1–Q3) age at inclusion date was 37.0 (25.0–48.0) years, with 69.5% of patients being female. The mean (SD) reimbursed cost over follow-up was €24,310 (89,167), mostly represented by hospitalization costs accounting for €21,156 (86,402). A switch in SCD management was observed with age, as younger patients presented more frequent hospitalizations and acute procedures, while older ones had more frequent medical visits and paramedical care. Mean (SD) annual costs were €25,680 (91,843) and vs. €3,227 (23,372) for patients and controls, respectively (*p* < 0.001), representing an extra cost of almost €150 million over the entire SCD population. This study highlighted the important costs related to SCD and the related medical need with treatment alternatives, which could be filled by the emergence of new therapies.

## Introduction

Sickle cell disease (SCD) is a rare genetic disorder characterized by a mutation in hemoglobin, the oxygen-carrying protein. The mutated protein causes the fragilization of red blood cells, leading to acute vaso-occlusive crises (VOCs) and ischemia (1–3). Over the past decade in France, the prevalence of SCD increased by ≈5% per year and was estimated around 26,000 patients in 2011 (4, 5).

Aside from acute VOC, SCD’s main chronic manifestations are hemolytic anemia and progressive organic failure due to capillary vessel damage. These manifestations significantly reduce life expectancy, when compared to the general population (6).

To date, there are limited curative treatments for SCD, with most therapies being symptomatic, aiming to decrease the risk of VOCs. The two treatments available for this indication are hydroxycarbamide, which is the gold standard for VOC prevention, and crizanlizumab (rarely used in France – not yet reimbursed) (7–9). SCD management also involves acute and chronic blood transfusions, to reduce the proportion of mutated blood cells or to prevent poorly tolerated anemias (10). Recently, voxelotor has introduced a new approach for the chronic management of SCD-related hemolytic anemia (11, 12). Hematopoietic stem cell transplantation (HSCT) remains the only curative procedure currently available but is rarely performed due to a lack of donors and compatibility issues (13, 14).

Due to the acute clinical events and chronic complications, SCD burden is important in terms of hospitalizations, treatments, and general management. A previous study based on French claims database up to 2011 estimated an annual average hospital cost of about €6,000 per SCD patient (5). This study aimed (i) to provide updated data on the main characteristics, management, and costs of SCD among patients aged ≥12 years in France and (ii) to compare those costs to that of the general population in order to assess SCD additional costs.

## Materials and methods

### General design

This was an observational, retrospective, cohort study performed among adolescent and adult patients with SCD between January 1st, 2016, and December 31st, 2018. These patients were matched to individuals from general population (referred to as “controls”) over the same period. This study used secondary healthcare consumption data from the French permanent representative 1/97th sample (EGB – *Echantillon Généraliste des Bénéficiaires*), based on healthcare claims from the French national health data system (SNDS - *Système National des Données de Santé*) (15).

### Study population

Patients aged 12 years and older, or reaching the age of 12 years, who presented at least one SCD-related marker during the selection period (2016–2018) were included. SCD identification criteria were the following: at least one hospitalization and/or long-term disease (LTD) status with a SCD diagnosis [D57 code “Sickle-cell disorders” from the International Classification of Diseases – 10th revision (ICD-10)] and/or a dispensation of SCD-specific hydroxycarbamide tablet (French specific package identifier) between 2016 and 2018. LTD is an administrative status corresponding to patients with a major chronic disease, entitling them to full medical expense coverage. Among them, patients with a sickle-cell trait diagnosis (ICD-10 code D57.3 “Sickle-cell trait”) and those with insurance coverage discontinuation were excluded.

The date of inclusion was defined according to the patient’s age and the date of the earliest identifiable SCD marker, using a historical period down to 2011. For patients aged ≥12 years as of January 1, 2016, with a history of SCD management, the inclusion date was January 1, 2016. For those aged ≥12 years as of January 1, 2016, with no history of SCD management, as well as those aged <12 years as of that date, the inclusion date was the date of the first sickle cell marker with appropriate age. Patients were described according to their age group at the date of inclusion, between teenagers [12–20 years], young adults [20–40 years], and older adults [≥40 years].

Sociodemographic and clinical characteristics were described at the inclusion date. Treatment history was assessed in the year preceding inclusion. Patients were followed for ≥6 months and up to the end of the extraction period (2018) or death, whichever occurred first.

SCD patients were matched on a 1:3 ratio to individuals with no diagnosis of SCD, comprising the control group. The matching was conducted through direct strict matching based on age, sex, CMU-c status (which corresponds to free-of-charge complementary coverage for people with low resources) and geographical region of residence. SCD patients and their matched controls were followed-up for the same duration by adjusting controls’ follow-up period to that of the corresponding patients.

### Data source

The French national claims database (*Système National des Données de Santé – SNDS*), exhaustively gathers the reimbursed healthcare resources of individuals covered by one of the compulsory healthcare plans, encompassing nearly 99% of French residents. The EGB is a 1/97th sample of ≈700,000 individuals extracted from SNDS. It is an exhaustive pseudonymized patient-level collection of claims data, representative of the French population in terms of age, gender, and geographical area. Healthcare reimbursements are recorded using specific coding systems for a diverse range of variables, including procedures, laboratory tests, medical devices, diagnoses (hospitalizations or LTD), or reimbursed drugs. Among other main data available, beneficiary information include age, gender, city of residence, date of care provision, care settings, as well as date of death.

### Outcomes and variables

The main outcome was healthcare consumptions and related costs among SCD cases alone and versus matched controls, overall and by healthcare resource type. Healthcare resource types included laboratory tests, paramedical care (comprising nursing care and physiotherapy sessions), overall treatments (versus controls) and treatments of interest (hydroxycarbamide, opioids, erythropoietic growth factors, iron chelators), medical visits, hospitalizations, imaging exams, procedures, and emergency room (ER) visits. Costs related to each of these items were also described.

The secondary outcomes were SCD patients’ sociodemographic and clinical characteristics, as well as main treatments of interest received.

### Statistical methods

Quantitative variables were described in terms of mean, standard deviation, median, first (Q1) and third (Q3) quartiles, and extreme values; qualitative variables in terms of absolute frequency and percentage by category. The description of healthcare resources used the proportion of patients with each relevant consumption and number per patient-years. Data from EGB were extrapolated to obtain estimates of patients, procedures, and costs on a national scale. Extrapolations were calculated for each year, adjusted on age and gender based on the French population census for the same year (16).

SCD attributable cost was calculated by comparing the SCD cohort and the control population as proportions of patients with at least one healthcare consumption of interest and costs assessment. Statistical tests were adapted according to the number of patients and the statistical distributions. Main tests used were Fisher exact test, Yate chi squared test and bootstrapped Student test.

All analyses were performed using SAS® version 9.4 (SAS Institute Inc. Cary, NC, USA).

### Ethical considerations

Before access to the data, the protocol was validated by an independent scientific committee and by the Health Data Hub as part of a simplified EGB procedure. Once approved, EGB data were analyzed on the secure SNDS portal by data managers and statisticians trained in patient data security standards. No individual data were extracted from the SNDS portal. This study was conducted in compliance with the French Data Protection Act and in accordance with applicable and with the ethical principles set out in the Declaration of Helsinki.

## Results

**Figure 1:**
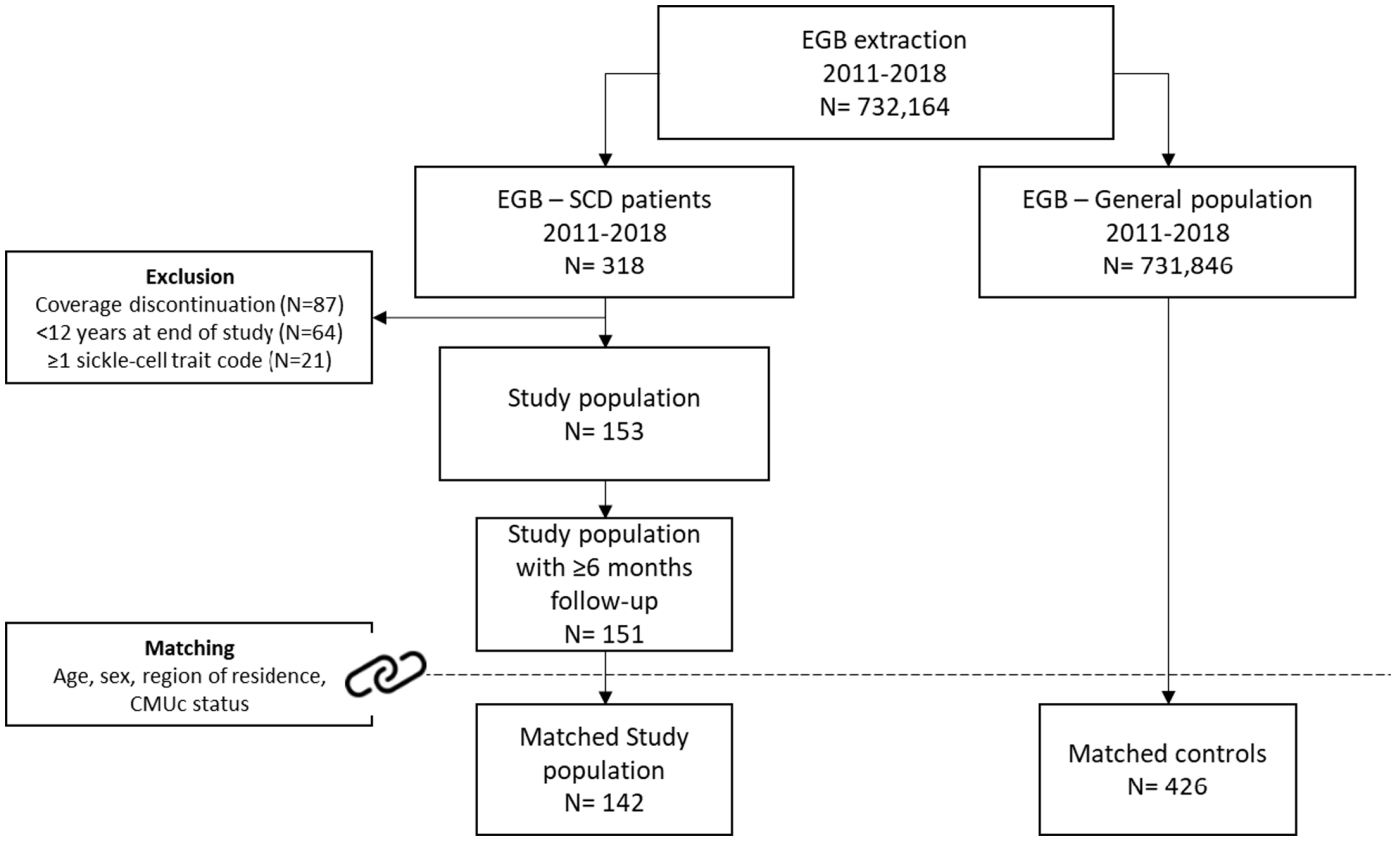
Patient disposition. CMUc corresponds to free-of-charge complementary coverage for people with low resources.

Among the 732,164 individuals present in the EGB, 318 were identified with at least one SCD marker during the inclusion period. Of them, 153 fulfilled selection criteria, and 151 had at least a 6-month follow-up period (Figure 1). The SCD prevalence extrapolated to the entire French population [95% CI] increased from 15,673 in 2016, to 19,502 patients [19,230; 19,778] in 2018.

### Patient characteristics

The median (Q1 – Q3) age at the inclusion date was 37.0 (25.0–48.0) years. Patients were mostly female (69.5%) and 84.0% of SCD patients lived in French mainland (*n* = 121), while the remaining 16.0%, lived in overseas regions (Table 1).

**Table 1:** Patient characteristics.

	Teenagers	Young adults	Older adults	Study population
	[12–20 years]	[20–40 years]	[≥40 years]	[≥12 years]
	(*N* = 25)	(*N* = 62)	(*N* = 64)	(*N* = 151)
**Age at inclusion**				
Mean (SD)	13.4 (1.7)	30.6 (5.6)	53.8 (11.8)	37.6 (17.4)
Median (Q1 – Q3)	13.0 (12.0 – 15.0)	31.5 (26.0 – 35.0)	51.0 (46.0 – 60.0)	37.0 (25.0 – 48.0)
**Sex**				
% women	52.0	79.0	67.2	69.5
**Treatments (%)**				
Opioids	40.0	51.6	39.1	44.4
Hydroxycarbamide	16.0	14.5	7.8	11.9
Transfusion	20.0	9.7	1.6	7.9

SD, standard deviation. Treatments identified within 1 year prior to inclusion date. Transfusion gathers both transfusions and aphereses.

During the year before inclusion, 7.9% of patients received a least one transfusion (encompassing both transfusions and aphereses), mostly among teenagers. Hydroxycarbamide use was identified in 11.9% of patients, with a slight decrease with age. Finally, nearly half (44.4%) of the patients received opioids during this period with little difference between age groups (Table 1). Finally, no HSCTs were identified, neither in the historical period nor in the follow-up period.

### Healthcare resource use

#### SCD-related HCRU and costs

Over the 36-month follow-up period, nearly every patient had at least one healthcare resource use (HCRU) of interest (*n* = 145, 96.0% – Table 2).

**Table 2:** Healthcare resources used and related costs.

	Teenagers	Young adults	Older adults	Study population
	[12–20 years]	[20–40 years]	[≥40 years]	[≥12 years]
	(*N* = 25)	(*N* = 62)	(*N* = 64)	(*N* = 151)
Healthcare resources used (rate per patient-years [95%CI])				
Laboratory tests	19.89 [18.79; 21.03]	41.89 [40.90; 42.89]	43.41 [42.42; 44.42]	39.07 [38.45; 39.69]
Paramedical care	1.99 [1.66; 2.38]	7.08 [6.68; 7.50]	16.74 [16.12; 17.37]	10.40 [10.09; 10.73]
Treatments of interest	2.28 [1.92; 2.69]	2.18 [1.96; 2.42]	3.02 [2.76; 3.29]	2.55 [2.40; 2.72]
Outpatient visits	1.32 [1.05; 1.64]	2.14 [1.92; 2.37]	3.37 [3.10; 3.66]	2.54 [2.38; 2.70]
Imaging exams	2.35 [1.98; 2.76]	1.40 [1.23; 1.60]	0.79 [0.66; 0.94]	1.29 [1.18; 1.41]
Emergency room visits	1.16 [0.91; 1.46]	1.32 [1.15; 1.50]	0.58 [0.47; 0.70]	0.97 [0.88; 1.08]
Procedures of interest	1.50 [1.21; 1.83]	1.51 [1.32; 1.71]	0.27 [0.19; 0.36]	0.97 [0.88; 1.08]
Hospitalizations	2.35 [1.98; 2.76]	2.11 [1.89; 2.35]	1.31 [1.15; 1.50]	1.81 [1.68; 1.95]
Costs (€) reimbursed by Health Insurance [mean (SD)]				
Laboratory tests	248 (262)	505 (806)	518.2 (579)	468 (652)
Paramedical care	88 (242)	391 (1,838)	1,101.0 (2,531)	642 (2,059)
Treatments of interest	3,355 (10,337)	1,740 (5,852)	1,872.4 (5,032)	2,064 (6,480)
Outpatient visits	100 (236)	121 (122)	218 (265)	158 (217)
Imaging exams	129 (192)	62 (126)	55 (85)	70 (127)
Emergency room visits	36 (39)	49 (86)	21 (33)	35 (63)
Procedures of interest	0 (0)	1 (7)	0 (0)	0 (5)
Hospitalizations	15,796 (27,339)	28,030 (127,537)	16,433 (38,205)	21,156 (86,403)
Total	17,973 (28,538)	30,976 (131,507)	20,330 (39,991)	24,380 (89,198)

SD, standard deviation. Treatments of interest (hydroxycarbamide, opioids, erythropoietic growth factors, iron chelators).

Over this period, the mean (SD) cost associated with HCRU of interest reimbursed by French Health Insurance was €24,310 (89,167). Most of this amount (87.0%) was represented by hospitalization costs, with €21,156.0 (86,403). Treatments of interest accounted for €2,064 (6,480). Overall, 97.0% of the costs were reimbursed by French Health Insurance.

Average costs were consistent among teenagers and older adults, with €17,841 (28,466) and €20,276 (39,989), respectively. However, a peak appeared among young adults [20–40 years], with €30,914 (131,461). Whatever the age group, around 90% of these costs were attributed to hospitalizations (Table 2).

#### Comparison with the control population

Among the 151 patients from the study population, 142 (94.0%) were successfully matched, leading to a control cohort comprising 426 individuals.

**Figure 2:**
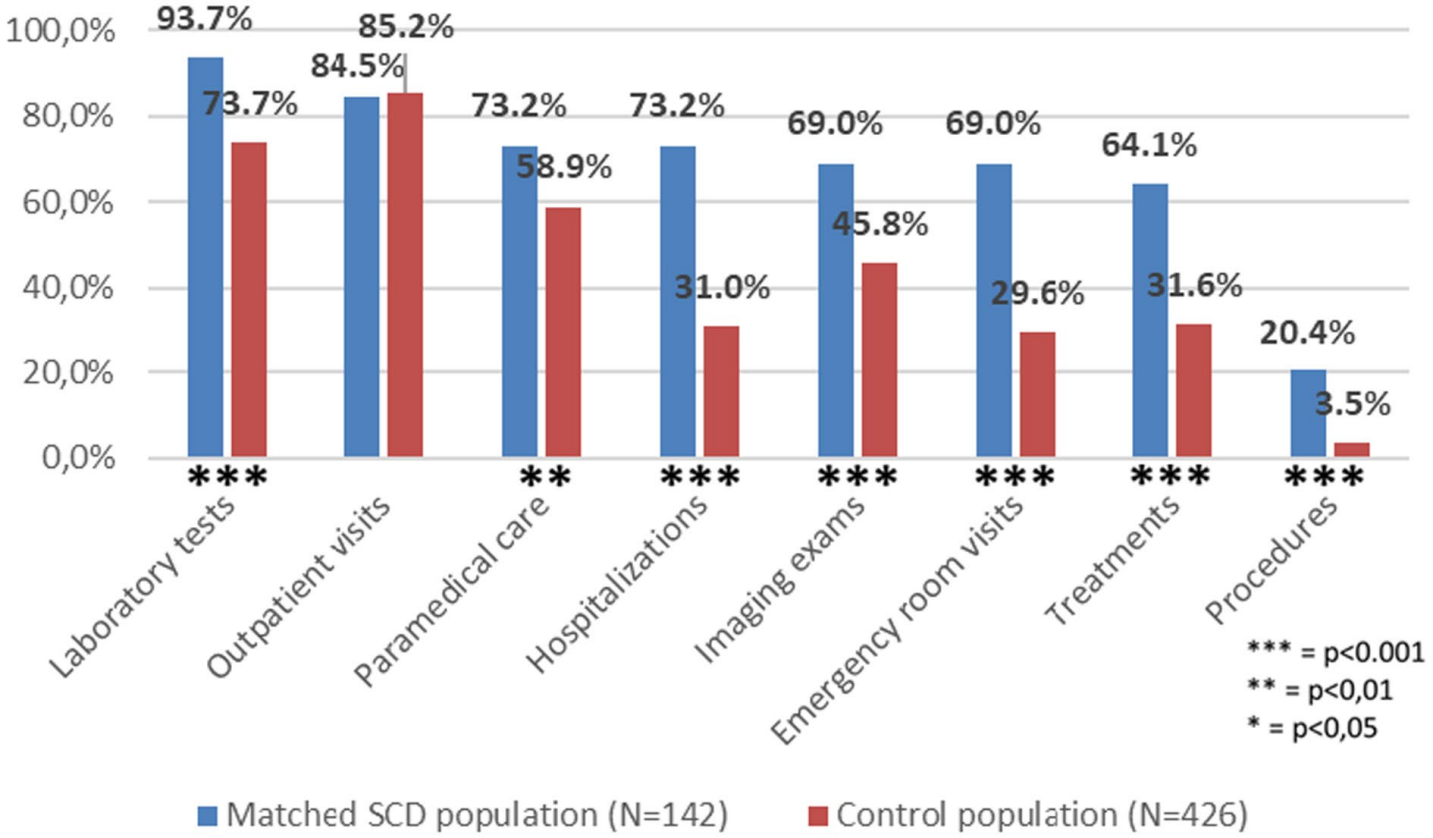
Comparison of proportion of individuals with at least one healthcare resource use of between matched SCD and Control populations.

In terms of the overall proportion of individuals with at least one HCRU of interest, no significant difference was observed between SCD patients and controls. Rates of patients and controls with at least one outpatient visit were similar with 84.5 and 85.2%, respectively. However, SCD patients displayed significantly higher proportions in all other HCRU categories. The most significant differences were found for procedures with 20.4% of patients and 3.5% of controls, respectively (*p* < 0.001); hospitalizations (73.2% versus 31.0%, *p* < 0.001); and ER visits (69.0% versus 29.6%, *p* < 0.001) (Figure 2).

The mean (SD) costs were 8 time higher in the SCD population compared to controls, €25,681 (91,844) versus €3,228 (23,373) respectively (*p* < 0.001). Hospitalizations accounted for over 90% of SCD-related costs and 80% of control-related costs, implying a significant contrast between SCD patients and controls, with means of €22,321 (89,005) and €2,556 (23,143), respectively, representing close to a ninefold increase. Overall, costs of treatments, procedures, paramedical care, outpatient visits, laboratory tests, imaging exams and ER visits amounted to €3,584 among SCD patients versus €644 for controls (*p* < 0.001). No significant difference was observed for procedures and outpatient visits (Table 3).

**Table 3:** Cost comparison between Matched SCD and Control populations.

Mean (SD) Costs (€)	Matched SCD population	Control population	Value of *p*
	(*N* = 142)	(*N* = 426)	
Age at matching			
Mean (SD)	37.4 (18.5)	37.4 (18.5)	/
Sex			
% women	68.9	68.9	/
Hospitalizations	22,321 (89,005)	2,556 (23,143)	***p* < 0.001**
Other costs	3,584	644	
Treatments	2,195 (6,663)	16 (166)	***p* < 0.001**
Paramedical care	615 (1,981)	227 (873)	***p* = 0.002**
Laboratory tests	495 (663)	187 (352)	***p* < 0.001**
Outpatient visits	167 (221)	178 (242)	*p* = 0.651
Imaging exams	75 (129)	25 (55)	***p* < 0.001**
Emergency room visits	37 (64)	11 (26)	***p* < 0.001**
Procedures	0 (5)	0 (0)	*p* = 0.090

SD, standard deviation. Figures in bold correspond to *p-*values < 0.05.

## Discussion

### Patient characteristics

With almost 20,000 patients with SCD estimated in 2018, the extrapolation of EGB data seems to be within the lower bound of the expected prevalence as reported in the literature. Leleu et al. study using French claims data projected SCD prevalence to range between 19,800 and 32,400 in 2016 (5). This lower-than expected prevalence could be due, in part, to the exclusion of patients with discontinued health insurance coverage in our study. Taking these patients in the assessment (87 patients excluded on this basis) could lead to a prevalence of more than 30,000 patients.

Despite being of recessive autosomal transmission, the rate of women with SCD seemed surprisingly high, notably among women of childbearing age (79%). This discrepancy could be partly explained by coding inaccuracies, especially among pregnant women with sickle cell trait. Often asymptomatic, these individuals may experience several complications during their pregnancy, leading to a hospitalization during which they could be incorrectly coded as having SCD. The absence of symptom could limit the likelihood of a contradictory diagnosis with sickle cell trait code, as highlighted by Leleu and Brousse studies (5, 17). The lack of precise genotyping in EGB (HbS/HbC etc.…) did not allow further differentiation between patients, according to severity or potential miscoding.

Regarding SCD treatments, HU remains the gold standard, but the proportion of patients treated with HU in this study seemed relatively low, with only 11.9% of SCD patients. As HU is always reimbursed, EGB is expected to provide comprehensive coverage of its utilization. This could highlight a potential discrepancy between prescriptions and actual dispensations. Reasons for such a low rate are multiple but ineffectiveness could be highlighted. It can be notably due to pharmacokinetic polymorphisms or concomitant Hb mutations such as α-thalassemia or HbF mutations [the latter being potentiated by HU (18)]. Additionally, poor treatment adherence is one of the most important and changeable factors to ensure treatment effectiveness (19). Several studies emphasized the lack of medication adherence among patients with SCD, as Zhou et al. claims study showing that the median adherence to HU varied between age classes, and was the lowest among young adults, with around 30% (20, 21). Due to the nature of the database, it was not possible to distinct cases when HU is not prescribed – reflecting prescription patterns – from cases when HU is prescribed but not dispensed – reflecting adherence itself. Sensitization of patients and practitioners on the role of HU in the prevention of SCD crises could help increasing the rate of patients treated with HU.

Despite its status as the sole curative therapy for SCD in France to date, no HSCT procedure has been recorded during the 3-year follow-up period of this study, despite an excellent long-term SCD-related event-free survival. This result can be explained by the rarity of HLA-matched donors, and the relatively limited size of the patient sample included in this study (14, 22).

### SCD overall costs

Unsurprisingly, healthcare consumptions seemed to increase with age in this study. This trend was mostly driven by laboratory tests and paramedical care. In the other hand, imaging exams were more frequently performed among teenagers. This is in line with SCD physiopathology, as the most frequent imaging exams are typically related to the spleen, gallbladder, and transcranial doppler assessments. Several studies showed that in the early years of life, children with SCD were at higher risk of strokes, transient ischemic attacks, or seizures, necessitating regular Doppler assessments at this age (23, 24). A similar – less pronounced – trend was observed for transfusions and ER visits. This tends to highlight a potential shift in symptomatology with advancing age, transitioning from acute events among young patients, to chronic forms and complications among older ones, associated with a shift from inpatient to outpatient care settings.

### SCD attributable costs

The last part of this study focused on comparing costs of SCD patients to those of the general population.

As anticipated, except for outpatient visits for which a similar proportion of individuals had at least one visit in both groups, significant differences were encountered for each other type of HCRU of interest.

Overall, mean costs among SCD patients were 8 times greater than those of general population. This is in line with Leleu et al. estimation with an annual cost per patient estimated around €6,000 in mean (5). Based on these results, the national extrapolation of SCD attributable cost would lead to a total cost of almost €450 million over the 3-year follow-up period, or €150 million annually. It is worth noting that this study excluded certain patient groups, including those with healthcare coverage discontinuation or late diagnoses. Leleu et al. estimated SCD prevalence in France between 19,800 and 32,400 patients. Should these excluded patients be considered, it could increase the total costs to over €190 million per year (5). Also, a considerable variability exists in SCD related costs between patients.

In this study, only direct medical costs were included, as part of the health insurance perspective. However, from a societal standpoint, EGB does not bring sufficient granularity to assess indirect costs such as productivity loss due to VOCs or caregiver burden, as SCD repercussions go way beyond this scope and strongly impact patients’ and caregivers’ daily lives, both in terms of activity and quality of life. In 2021, Holdford et al. conducted a survey among 192 SCD patients, of whom 187 reported a vaso-occlusive event. Results showed that absenteeism related to pain events incurred an average annual cost of $15,000 per patient. Total annual losses in unpaid work productivity averaged $3,145,862 for the study respondents and another $2,870,652 for their caregivers (25). Additionally, Adam et al. demonstrated a significant prevalence of depression among SCD patients, with 35.2% of patients either treated or showing depressive symptoms and an overall decrease in quality of life, according to SF-36 scale, potentiated by depression (26). Aside from the differences in healthcare access between France and the United Stated where these two studies come from, this highlights the importance of non-medical and indirect costs in the exhaustive assessment of the burden of a chronic disease such as SCD.

Finally, a substantial proportion of SCD patients did not receive any chronic treatment or were ineligible to HSCT. Furthermore, and based on the overall improvement in the management and prevention of SCD acute events and complications, notably among young children with vaccinations and prophylactic antibiotherapy, more and more patients are expected to reach advanced ages, as shown by the increase of life expectancy among SCD patients over the past decades. According to these statements, and with no new treatment available, SCD clinical and economic burden should progressively increase in the years to come, with an increasing number of patients requiring invasive and expensive procedures such as dialyses or organ transplants.

### Strengths and limitations

Despite the granularity of data available in the EGB database, some pieces of data are not available. For instance, stays in post-operative rehabilitation centers and psychiatry centers data cannot be captured. Even with the majority of SCD management originating from hospitalizations and treatments, leading to expect this bias to be limited, a potentially non-negligible part of SCD related costs might have been underestimated due to the absence of rehabilitation care data, notably for patients experiencing a stroke or any other highly debilitating complication. For instance, in general population, Gabet et al. showed that more than one third of patients experiencing a stroke were referred to a SSR structure with lengths of stays varying between 36 and 60 days in median (27).

Another unavailable source of data is the drugs paid over the counter (OTC). These OTC drugs are mostly painkillers or symptomatic treatments. In addition to representing a small cost, most of these drugs could be 100% reimbursed when prescribed by a general practitioner, hence detected in this study, leading to a very low impact.

As SCD remains a rare disease, with non-specific clinical manifestations, the development of tailored algorithms for SCD patient identification remains a potential source of bias. The presence of ICD-10 codes D57 and specific treatments bring an important but non-exhaustive capture of SCD patients. Symptomatic patients are expected to be easily detected, while pauci-symptomatic ones are more prone to not be. This could lead to a selection bias and a slight overestimation of SCD burden, as more severe patients are analyzed.

The incomplete matching ratio (94.0%) can be explained by the choice of strict variables for direct matching (exact same age, region of residence…). However, overall costs from the study population and the matched SCD subgroup remained similar in mean [95%CI] (€24,380 [13,593; 41,026] versus €25,681 [14,407, 43,583]).

Also, a potential lack of power can be identified with EGB data, as the number of patients with rare disease could lead to small samples making result generalization more difficult. Concerning this limitation, a recent study by Brousse et al. based on the complete extraction of SNDS data showed similar results regarding sociodemographic and clinical results, with similar age distribution among patients aged 12 years and older, and similar rates of acute and chronic complications such as infections and chronic kidney disease. This emphasizes the potential generalizability of EGB data to the French population. The comparison of costs between the studies tended to highlight the importance of SCD related cost, overall, and notably among younger patients. In fact, the mean annualized costs among SCD patients according to Brousse et al. was of €16,000, mostly represented by hospital costs, while it was around €8,000 in our study. With patients aged less than 12 years representing more than 30% of the population in Brousse et al. study, an important part of overall SCD burden could be attributable to younger patients (17).

Nonetheless, and despite the biases and limitations identified above, this study’s methodology and data depth allow to depict a quite exhaustive and realistic overview of SCD public health impact among patients aged 12 years of older in France. To the best of our knowledge, it is one of the first study to bring real-world data on SCD attributable cost in France.

## Conclusion

This study estimated the SCD prevalence in France between 2016 and 2018 to be of more than 19,000 patients. A change in the needs of SCD patients with age – reflected in this study by a switch in SCD management pattern – was observed. Younger patients had more frequent hospitalizations and acute procedures. In contrast, older ones have more frequent medical visits and paramedical care. Finally, this study showed the importance of SCD management and related costs, with HCRU-related costs being 8 times greater than the general population, representing a potential annual mean cost of more than €150 million. Future similar studies should be carried out to accompany the emergence of new therapies and assess potential public health benefits that could help tackle this important medical need. Finally, strategies to raise awareness among prescribers and patients of the importance of a standard-of-care therapies (such as hydroxycarbamide) in SCD remain a key to improving treatment adherence.

## Data availability statement

The data analyzed in this study is subject to the following licenses/restrictions: As per Decree 2021–848 du 29 juin 2021, data from SNDS which have been beforehand granted by an ethical and scientific validation committee can only be accessed by authorized companies. Requests to access these datasets should be directed to not applicable. For more information, see Health Data Hub website https://www.health-data-hub.fr/.

## Ethics statement

In accordance with the simplified procedure (deliberation 2020–072 of July 16, 2020 – CNIL), this study was validated by an independent scientific committee before access to the data. It was then approved by the HDH (n°4,894,596) on July 11, 2021, and was the subject of an agreement with the French health insurance system (Cnam) on October 19, 2021. No informed consent is required in accordance with decree n° 2021–848 of June 29, 2021, of the public health code.

## Author contributions

MB, LC, and ID-Z: manuscript development. SB and LC: statistical analysis. MB, SB, and FG: administrative, technical, or material support. SB: full access to all study data and takes responsibility for data integrity and accuracy of data analysis. MB, ID-Z, FP, SB, LC, and FG: critical revision of the manuscript for important intellectual content, supervision, concept and design, data acquisition, analysis, and interpretation. All authors participated in the interpretation of the data, provided critical feedback and final approval for submission, and took responsibility for the accuracy, completeness, and adherence to the data and analysis protocol.

## Funding

The study was funded by GBT-Pfizer. The funder had the following involvement with the study: concept and design, administrative support and supervision, decision to publish and preparation of the manuscript.

## Conflict of interest

MB is employed by Pfizer. ID-Z is Chair of the scientific committee of the French blood establishment and received Honoraria for GBT-Pfizer, Takeda, and Vifor. FP received Honoraria for Novartis, Celgene, Diagast, Gilead, Grifols, Addmedica, and GBT-Pfizer. SB is the director of stève consultants, which has a research consultancy contract with Pfizer. LC is employed by stève consultants, which has a research consultancy contract with Pfizer. FG received Honoraria for Vertex, Novartis, Addmedica, GBT-Pfizer, and Agios.

## Publisher’s note

All claims expressed in this article are solely those of the authors and do not necessarily represent those of their affiliated organizations, or those of the publisher, the editors and the reviewers. Any product that may be evaluated in this article, or claim that may be made by its manufacturer, is not guaranteed or endorsed by the publisher.
