# Supplementary material for: Prevalence and cost of sickle cell disease in France: real-world analysis using data from the Echantillon Généraliste des Bénéficiaires
Source: Front Public Health. 2023 Sep 21;11:1215605. doi: 10.3389/fpubh.2023.1215605 (PMC10552641; doi:10.3389/fpubh.2023.1215605)
Supplement: Supplementary file 1 [file Table_1.DOCX]

**Annexes**

Comorbidities and Medical events

Comorbidities

Each comorbidity of interest listed below will be looked for in PMSI based on ICD-10 codes, and/or as LDD list when available. PMSI researches will include:

- PMSI-MCO: DP, DR, DA of RUM or stays
- PMSI-HAD: DP, DCMPP, DCMPA

Table 1. Identification of Comorbidities

|  | **Algorithm (Codes in bold are groups of ICD-10 codes)** |
| --- | --- |
| Diabetes (uncomplicated) | Patients with at least one hospitalization diagnosis (PMSI) and/or LDD with an ICD-10 code from the following list:   - **E138 – E141;** E146 ; E148 ; E149   AND/OR  At least 3 reimbursements at different dates (or 2 in large packaging) of antidiabetic drugs:   - A10A ; A10B |
| Diabetes (complicated) | Patients with at least one hospitalization diagnosis (PMSI) and/or LDD with an ICD-10 code from the following list:   - **E102 – E105;** E107; **E112 – E115;** E117; **E122 – E125;** E127; **E132 – E135;** E137; **E142 – E145;** E147   AND/OR  At least one procedure for laser treatment of diabetic retinopathy (CCAM)   - **BGNP001 – BGNP008**, except BGNP002 and BGNP005 |
| Chronic respiratory diseases | Patients with at least one hospitalization diagnosis (PMSI) and/or LDD with an ICD-10 code from the following list:   - I278 ; I279; **J40 – J47; J60 – J67;** J684; J701; J703   AND/OR  At least 2 reimbursements (at different dates) for ATC **R03** class drugs |
| Heart failure | Patients with at least one hospitalization diagnosis (PMSI) and/or LDD with an ICD-10 code from the following list:   - I110; I130; I132; I50 Heart failure |
| Cancer | Patients with at least one hospitalization diagnosis (PMSI) and/or LDD with an ICD-10 code from the following list:   - **C00 – C26; C30 – C34; C37 – C41;** C43; **C45 – C58; C60 – C76; C81 – C85;** C88; **C90 – C97** |
| Cardiac arrhythmia | Patients with at least one hospitalization diagnosis (PMSI) and/or LDD with an ICD-10 code from the following list:   - I44; I45; I47; I48; I49 |

Charlson Comorbidity Index

Table 2. Identification of Charlson Comorbidity Index (CCI)

|  | **ICD-10 codes (hospitalisation diagnosis or LDD)** | **Medical acts** | **Drug** |
| --- | --- | --- | --- |
| Myocardial infarction | I21;I22;I252;I255 |  |  |
| Heart Failure | I110;I130;I132;I50 |  |  |
| Peripheral vascular pathology | I70;I71;I731;I738;I739;I771;I790;I792;K551;  K558;K559; Z958;Z959 | Peripheral stent placement |  |
| Cerebrovascular pathology | G45;G46;H340;I60-I69 |  |  |
| Dementia | F00-F03;F051;G30;G311 |  | At least 3 reimbursements for medication indicated for Alzheimer's disease (N06DA, N06DX01) |
| Chronic Pulmonary Disease | I278;I279;J40-J47;J60-J67;J684;J701;J703 |  | At least 2 reimbursements for bronchodilator (R03) |
| Connectivity | M05;M06;M315;M32;M33;M34;M351;M353;M360 |  |  |
| Ulcer pathology | K25-K28 |  |  |
| Mild liver disease | B18;K700-K703;K709;K713K715;K717;K73; K74;K760;K762-K764;K768;K769;Z944 E100;E101;E106;E108;E109;E110;E111;E116; E118;E119; E120;E121; E126;E128-E131;E136 |  |  |
| Uncomplicated diabetes | E138-E141; E146;E148; E149 |  | At least 3 reimbursements (or 2 in large packaging) of antidiabetic drugs (ATC code: A10A and A10B) |
| Hemiplegia | G041;G114;G801;G802;G81;G82; G830; G831-G834;G839 |  |  |
| Moderate to severe kidney disease | I120;I131;N032-N037;N052-N057;N18;N19;  N250;Z490;Z491; Z492;Z940;Z992 | Dialysis session |  |
| Diabetes with complication | E102-E105;E107;E112-E115;E117;E122-E125; E127;E132-E135;E137; E142-E145;E147 | Laser treatment of diabetic retinopathy |  |
| Cancer (including lymphomas and leukemias and excluding neoplastic skin pathologies) | C00-C26;C30-C34;C37-C41;C43; C45-C58; C60-C76;C81-C85;C88; C90-C97 |  |  |
| Moderate to severe liver disease | I850;I859;I864;I982;K704;K711;K721;K729; K765-K767 |  |  |
| Metastatic disease | C77-C80 |  |  |
| HIV-AIDS | B20-B22;B24;Z21 |  |  |

Acute SCD-related events

Table 3. Identification of patients with acute SCD-related events

|  | **Algorithm** |
| --- | --- |
| Acute SCD episode requiring hospitalization | At least one hospitalization diagnosis – **PMSI-MCO** DP, DR with ICD-10 code:   - D57.0; D57.1; D57.2; D57.8 |
| Myocardial infarction (included in VOC) | At least one hospitalization diagnosis – **PMSI-MCO** DP, DR with ICD-10 code:   - I21; I22 |
| Stroke | At least one hospitalization diagnosis – **PMSI-MCO** DP, DR with ICD-10 code:   - G45; G46; H340; **I60 – I66** |
| Acute Spleno-biliary events | At least one hospitalization diagnosis – **PMSI-MCO** DP, DR with ICD-10 code:   - D73.1; D73.5; D73.8; D73.9; K80; K81.0; K81.8; K82; K83.1; K83.8 |
| Acute Hepatic events | At least one hospitalization diagnosis – **PMSI-MCO** DP, DR with ICD-10 code:   - K72.0; K72.9; K76.3; K76.5; K75.1; K76.2; K76.8; K77.8 |
| Acute Renal events | At least one hospitalization diagnosis – **PMSI-MCO** DP, DR with ICD-10 code:   - N00; N10; N16; N17; **N20 – 23** |
| Acute renal failure | At least one hospitalization diagnosis – **PMSI-MCO** DP, DR with ICD-10 code:   - N17 Acute renal failure |
| Severe infection – sepsis | At least one hospitalization diagnosis – **PMSI-MCO** DP, DR with ICD-10 code:  A02.1; A20.7; A21.7; A22.7; A24.1; A26.7; A32.7; A39.2; A39.3; A39.4; A39.5; A40; A41; A44.0; A48.3 ;A42.7; B00.7; B37.7 ;B38.7 ;B39.3; B40.7; B41.7; B42.7; B44.7; B45.7; B46.4; O85; R57.2; R65.1 |
| Severe infection – pneumonia | At least one hospitalization diagnosis – **PMSI-MCO** DP, DR with ICD-10 code:  A15; A16; A20.2; A21.2; A22.1; A31.0; A42.0; A43.0; A48.1; B01.2; B05.2; B25.0; B37.1; B38.0; B38.1; B38.2; B39.0; B39.1; B39.2; B40.0; B40.1; B40.2; B41.0; B42.0; B44.0; B45.0; B46.0; B59; J09; J10; J11; J12; J13; J14; J15; J16; J17; J18; J20; J85.1 ;J96.0 |
| Priapism | At least one hospitalization diagnosis – **PMSI-MCO** DP, DR with ICD-10 code:   - N48.3 |

Complications

Table 4. Identification of patients with SCD-related complications

|  | **Algorithm** |
| --- | --- |
| Pulmonary hypertension | Patients with at least one hospitalization diagnosis (PMSI) and/or LDD with an ICD-10 code from the following list:   - I27.0; I27.2 |
| Chronic Renal affections | Patients with at least one hospitalization diagnosis (PMSI) and/or LDD with an ICD-10 code from the following list:   - I120; I13; K767; N03; N04; N05; N11; N12; N16; N18; N19; N25 |
|  | Patients with at least one hospitalization diagnosis (PMSI) and/or LDD with an ICD-10 code from the following list:   - Y84.1; Z99.2   AND/OR  At least one medical procedure for which the CCAM code belongs to the following list:   - JVJB001 ; JVJF004 ; JVJF008 ; JVRP004; JVRP007 ; JVRP008 ; YYYY007   AND/OR  At least one of the following GHM codes:   - 11K021 ; 11K022 ; 11K023 ; 11K024 ; 11K02J ; 28Z01Z ; 28Z02Z ; 28Z03Z ; 28Z04Z ; 28Z05Z ; 28Z06Z   AND/OR  At least one of the following dialysis allowances:   - [At least one *Code de la Prestation de référence* (i.e. variable PRS_NAT_REF) of interest:   - 2121 ; 2122 ; 2123 ; 2124 ; 2125 ; 2126; 2127 ; 2128 ; 2129 ; 2131 ; 2132 ; 2134 ; 2135 ; 2136 ; 2137 ; 2138 ; 2139 ; 2140 ; 2142 ; 2143 ; 2144 ; 2145 ; 2146 ; 2147 ; 2334 ; 2163 - At least one fee (i.e. variable GHS_NUM) (PMSI-MCO) of interest:   - **D01–D24** - At least one hospital stay (PMSI-MCO) with:   - SUP_ENT_DPA =1   - And/or SUP_ENT_DPC = 1   - And/or SUP_ENT_HEM = 1 - And/or SUP_HEM_HS = 1 |
| Osteomyelitis | Patients with at least one hospitalization diagnosis (PMSI) and/or LDD with an ICD-10 code from the following list:   - H702 ; M462 ; M86 |
| Osteonecrosis | Patients with at least one hospitalization diagnosis (PMSI) and/or LDD with an ICD-10 code from the following list:   - M87.0; M87.3; M87.8; M87.9; M90.4; M90.5 |
| Leg ulcers | Patients with at least one hospitalization diagnosis (PMSI) and/or LDD with an ICD-10 code from the following list:   - I83.0; I83.2; L97 |
| Cellulitis | Patients with at least one hospitalization diagnosis (PMSI) and/or LDD with an ICD-10 code from the following list:   - L03; N730; N731; N732 |
| Retinal disorders | Patients with at least one hospitalization diagnosis (PMSI) and/or LDD with an ICD-10 code from the following list:   - H36.8 |

Procedures of interest

Table 5. CCAM codes for procedures of interest

| **CCAM codes** |
| --- |
| Cholecystectomy : HMFC004; HMFA007; HMFA001; HMFC005; HMFA005; HMFA004; HMFA008; HMFC001; HMFC003; HMFC002; HMFA002; HMFA006; HMFA003 |
| Phlebectomy : FEJF003 |
| Splenectomy : FFFA001; FFFC001; FFFA002; HNFC002; HNFA013; HNFA004; HNFA006 ;HNFA010; FFFC420; EHCA007 |
| Dialysis: JVJB001; JVJF004 ; YYYY007 |
| Hematopoietic Stem Cell Transplantation (HSCT): FELF009 |
| *Simple blood transfusions: FELF011; FELF006; FELF003 ;FELF004; FELF008 ;FEJF006* |
| ***Apheresis: FEPF005; FEPF003; FELF012*** |
| Hip/shoulder prosthesis: NEMA011; NEKA018; MEKA008; MEKA005; MEMA009; MEKA001 |
| Tonsillectomy FAFA005; FAFA006; FAFA010; FAFA014; FAFA015 |

Table 6. Codes for other transplantations

| Algorithm |
| --- |
| Other transplantations: At least one hospitalization diagnosis – PMSI-MCO DP, DR with ICD-10 code Z945; Z946 |

Table 7. Codes for oxygenotherapy

| Algorithm |
| --- |
| At least one of the following CCAM codes  GLLD019; GLLD003; GLLD006; GLLD013; GLLD002; GLLD012; GLLD015; GLLD008; GLLD004; GLLD017; GLQP001; GLQF001; GLMF001 ;GLMP001; GLLP003; GLKP001  AND/OR  At least one of the following LPP codes  1130220; 1191568; 1143983; 1149106; 1118324; 1174133 ;1125100; 1138315; 1105528 ;1151942; 1116680; 1105416; 1123609; 1186685; 1103297 ;1119789 ;1120338; 1184315; 1133430 ;1136581 ;1148130; 1165944; 1136227; 1128104  AND/OR  At least one hospitalization diagnosis – **PMSI-MCO** DP, DR, DAS with ICD-10 code Z991  AND/OR  At least one of the following codes PRS_NAT / DDP_COD  3511; 645; 646 |

Table 8. Other medications of interest

| **Name** | **ATC/CIP code** |
| --- | --- |
| Hydroxycarbamide (all forms) | L01XX05 |
| Siklos® | 3400938101942  3400941580574 |
| Hydrea® | 3400930512685 |
| Hydroxycarbamide | 3400937078528 |
| Opioids and other type II/III analgesics | N02A |
| Iron chelators | V03AC |
| Erythropoiesis stimulating agents | B03X |
